# Pilot of Direct Observation of Clinical Skills (DOCS) in a Medicine Clerkship: Feasibility and Relationship to Clinical Performance Measures

**DOI:** 10.3885/meo.2009.T0000137

**Published:** 2009-08-05

**Authors:** Yoon Kang, Charles L. Bardes, Linda M. Gerber, Carol Storey-Johnson

**Affiliations:** Weill Cornell Medical College, New York, New York

## Abstract

**Purpose:**

To assess the feasibility of Direct Observation of Clinical Skills (DOCS), a program for formative assessment of students’ clinical skills during a medicine clerkship and to determine relationships between DOCS measures and other clinical performance measures.

**Method:**

From August, 2004 through June, 2005, Medicine Clerks assigned to the primary on-campus clinical site were asked to participate in the pilot phase of the DOCS program. Students were asked to complete at least one DOCS exercise focused on interviewing, physical examination, or oral case presentation.

**Results:**

Of the 79 students who rotated on the Medicine Clerkship during the pilot period, 79% (n = 62) participated in DOCS, and 163 forms were submitted for evaluation. Seventy-seven percent (77%) of the clinical observations occurred while on-call or during daily rounds. Seventy-three (73%) of observations were completed in 30 minutes or less. In 89% of encounters students received at least 5 minutes of verbal feedback. Satisfaction ratings from both students and observers were “moderately satisfied” or better. Global ratings from DOCS physical exam and case presentation sections were strongly correlated with both faculty ratings of clinical performance and final clerkship grade. DOCS measures were not statistically related to clerkship written examination scores.

**Conclusions:**

These data support the feasibility of the DOCS session for formative assessment of student interviewing, physical examination, and oral case presentation skills during a medicine clerkship. Observer ratings from DOCS physical examination and case presentation sections were found to be predictors of final clerkship grade.

Both the public sector and medical education accrediting and licensure bodies have emphasized the importance of the demonstration of core clinical skills by learners.^1^,^2^ Direct observation and concurrent feedback is recognized as an important part of developing clinical skills^3^; students must now demonstrate competency in communication, interviewing, and physical examination as a requirement for medical licensure.

In stating the educational objectives of the Educational Program for the M.D. Degree, The Liaison Committee on Medical Education (LCME) asserts Standard ED-27: “*There must be ongoing assessment that assures students have acquired and can demonstrate on direct observation the core clinical skills, behaviors, and attitudes that have been specified in the school's educational objectives*.”^4^ However, it is widely recognized that medical schools often fall short in fulfilling this objective.^5^

An ongoing challenge for educational activities in the clinical setting is the increasing time constraint on clinical faculty and residents resulting from an evolving healthcare delivery system and residency training environment. Several studies document that direct observation as a means of assessing medical student clinical performance occurs infrequently^1^,^6^ and that when these observations occur they are primarily conducted by residents rather than by faculty^1^,^6^,^7^.

Barriers to direct assessment of clinical skills include pedagogic habits based on case discussion, the frequent omission of explicit criteria for evaluating clinical skills, time constraints, increased demands placed on clinical educators, decreased support for graduate medical education, and a host of other impediments. The biggest overall challenge is to balance two opposing forces: the ideal of frequent, real-time, validated assessment by faculty skilled in evaluation and feedback, and feasibility constraints of living and working in a busy medical center.

To meet the LCME standard while balancing the ideal and the practical, our medical college developed a curricular enhancement for evaluating clinical skills in the clerkships. The centerpiece of the program is called the DOCS session: Direct Observation of Clinical Skills. The purpose of this paper is to describe the implementation of DOCS and the evaluation of feasibility and the correlations between DOCS measures and other clerkship measures of student clinical performance.

## Developing the DOCS Session

The Dean charged the Clinical Curriculum Committee, a standing committee of the Medical College, with the task of designing programs that would fulfill LCME standards pertaining to the clinical curriculum. The Committee addressed Standard ED-27 in the following steps, undertaken over several months:

1. The Committee discussed and clarified the specific requirements of Standard ED-27.
2. Members of the Committee reviewed the current literature regarding the observation and assessment of clinical skills and then presented summaries to the group.
3. A member of the Committee, the Director of Standardized Patient Programs, created a draft instrument and presented it to the full Committee for discussion, modification, and ultimate adoption as the DOCS session.

The Direct Observation of Clinical Skills (DOCS) session and DOCS assessment forms were developed as a curricular enhancement to provide formative assessment of clinical skills and immediate, structured feedback. The sequential acquisition of clinical skills is evaluated through direct observation by standardized patients, faculty, and residents throughout our curriculum; the DOCS session is a component of this longitudinal assessment program.

The DOCS program focuses on clinical skills that could practically be assessed in the context of a clinical clerkship and includes three domains: interviewing, physical examination, and oral case presentation. DOCS consists of three separate exercises in which students are observed by either faculty or residents as they conduct a focused or complete history, perform a focused or complete physical examination, or present a patient's case orally. In order to construct the DOCS session as time-efficient observations that could be integrated into clinical work flow, the three observations do not need to be performed by the same observer or at the same time.

The American Board of Internal Medicine (ABIM) mini-clinical evaluation exercise (mini-CEX) form has been used successfully at other institutions for formative assessment in a medicine clerkship^7^,^8^. The mini-CEX form, however, was originally designed for direct observation of internal medicine residents. It evaluates seven competencies using a nine-point scale. We designed assessment forms customized to the goals of our DOCS session. There is a separate form for interviewing, physical examination, and oral case presentation. Each of the three assessment forms (interviewing, physical examination and oral presentation) is composed of a checklist of core skills and a global rating scale to assess overall observed performance.

Because the clinical approach of third year clerks is likely at the novice level in the Dreyfus model of skills acquisition^9^, we included checklists that emphasized specific elements of a clinical encounter and completion of core tasks on the DOCS forms. The DOCS checklists include 6–8 key steps in interviewing, physical examination, and oral presentation. Sample items from the interviewing checklist include the following: “Greeted patient and explained the purpose of the encounter” and “Accurately obtained relevant components of the history.” Both the format and content of the DOCS checklists are consistent with other instruments used to assess clinical performance in other parts of the curriculum, permitting potential comparisons.

The DOCS forms also include an overall global rating on a 1–5 scale. The DOCS global rating scale is consistent with the monthly evaluation forms used on the clerkship that employ the same numeric scale and descriptors. The global rating scale allows the observer to incorporate qualitative dimensions and nuances of observed performance that cannot be translated into a checklist item into their overall impression.

Additional data collected on the forms includes information about the observer (attending/resident), clinical setting in which the DOCS session was conducted, student and observer satisfaction with the DOCS form, and observation and feedback time. Observers and students complete and review the form at the time of the DOCS exercise, and students return the form in an attached self-addressed envelope.

Students learn about the DOCS session at the clerkship orientation, during which we discuss the purpose of the program, distribute an information sheet and the DOCS forms, and explain the logistics. The DOCS program is presented as a formal opportunity for students to receive feedback from either a resident or attending physician on observed clinical skills. We also explain the program and distribute DOCS forms to residents and attending physicians at the first morning report of each new clinical block. Students and observers are both asked to review the DOCS forms in order to set common expectations.

## Pilot Testing DOCS in the Medicine Clerkship

The Medicine Clerkship at our medical college is a 12-week course taken in the third year. It consists of three blocks lasting four weeks each: general and specialty medicine at the main teaching hospital, intensive care and cardiology in the main hospital, and general medicine at a community-based affiliate hospital.

Clerkship grades are determined by a consensus process that involves the course and site directors. The components included in evaluating student performance are a summary of clinical ratings from faculty and residents who work with the students at multiple clinical sites and a written exam score. Potential grade assignments for students who complete all course requirements are Honors, High Pass, Pass, Marginal, and Fail.

During the pilot test conducted from August, 2004 through June, 2005, Medicine Clerks were asked to complete DOCS sessions devoted to interviewing, physical examination, and oral case presentation. In order to provide a truly formative opportunity, students completed DOCS sessions and submitted DOCS assessment forms prior to week 9 of the 12-week clerkship. The results had no bearing on summative assessment and did not affect grades.

The study was approved by the Weill Cornell Medical College Institutional Review Board.

## Results

Relationships between DOCS measures and clerkship performance measures, including faculty and resident ratings of student clinical skills, written exam score and summary scores, were examined using Spearman's correlation coefficient. Fisher's Exact Test was used to evaluate the association between the number of forms returned per student and the student's final grade. Two-tailed probability levels for statistical significance tests are reported. Analyses were performed in SAS Version 9.1 (SAS Institute, Inc., Cary, North Carolina).

Of the 79 students rotating on the Medicine Clerkship during the pilot period, 79% (n = 62) participated in DOCS. One hundred sixty-three forms were submitted. The overall return rate for the DOCS forms was 70%. Table 1 Presents the clinical setting and type of observer completing the DOCS forms. The setting of the direct clinical observations was primarily while on-call (48%) or during daily rounds (29%). Fifty-five faculty attendings and residents participated as evaluators. Residents completed 84% of the evaluations.

**Table 1.** The Clinical Setting and Observer of DOCS

	Interviewing/Communication	Physical Examination	Oral Presentation	Total
Clinical setting: N(%)	52 (33)	53 (33)	54 (34)	159 (100)
Daily Rounds: N(%)	10 (6)	11 (7)	25 (16)	46 (29)
Post-call Rounds: N(%)	0 (0)	0 (0)	3 (2)	3 (2)
On-call: N(%)	34 (21)	32 (20)	11 (7)	77 (48)
Tutor Session: N(%)	2 (1)	2 (1)	8 (5)	12 (8)
Teaching Session: N(%)	3 (2)	4 (3)	4 (3)	11 (7)
Other: N(%)	3 (2)	4 (3)	3 (2)	10 (6)
				
Observer: N	51 (32)	55 (34)	54 (34)	160 (100)
Ward Attending: N(%)	0 (0)	0 (0)	1 (1)	1 (1)
Teaching Attending: N(%)	0 (0)	1 (1)	2 (1)	3 (2)
Tutor: N(%)	6 (4)	5 (3)	10 (6)	21 (13)
Chief Resident: N(%)	0 (0)	1 (1)	0 (0)	1 (1)
Resident: N(%)	45 (28)	48 (30)	41 (26)	134 (84)

Of the 145 DOCS sessions for which observation time was recorded, 73% were completed within 30 minutes with a mean time of 33 minutes. Feedback time was recorded for 147 encounters; 89% of these included at least 5 minutes of feedback, 74% included 5–15 minutes of feedback, and the mean amount of feedback time was 12 minutes. Table 2 Presents the observation and feedback times in more detail.

**Table 2.** Observation and Feedback Times for DOCS

	Interviewing/Communication	Physical Examination	Oral Presentation	Total
Observation Time: N = 145				
< 15 min: N(%)	5 (3)	8 (6)	14 (10)	27 (19)
15 – 30 min: N(%)	27 (19)	32 (22)	20 (14)	79 (54)
> 30 min: N(%)	15 (10)	12 (8)	12 (8)	39 (27)
Feedback Time: N = 147				
< 5 min: N(%)	4 (3)	6 (4)	6 (4)	16 (11)
5 – 15 min: N(%)	37 (25)	38 (26)	34 (23)	109 (74)
> 15 min: N(%)	5 (3)	7 (5)	10 (7)	22 (15)

**Figure 1.**
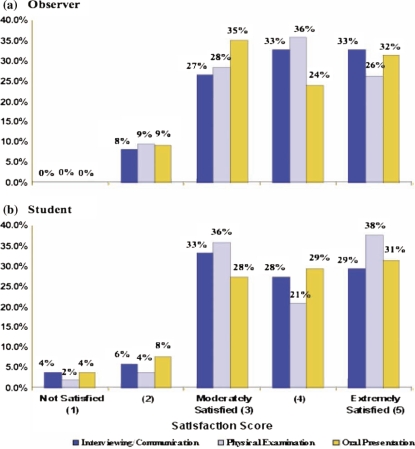
Distribution of DOCS Forms Satisfaction Ratings for Observers and Students, N for Observer Satisfaction Ratings= 163 (Interview = 52, Physical = 55, Oral Presentation = 56), N for Student Satisfaction Ratings= 157 (Interview = 51, Physical= 53, Oral Presentation= 53).

Figure 1 presents the satisfacation ratings for observers and students. Overall satisfaction with each of the DOCS forms (interviewing, physical examination, oral presentation) was rated from 1 (not satisfied) to 5 (extremely satisfied) on a Likert scale. Ninety-one percent (91%) of observer ratings and 90% of student ratings fell into the “moderately satisfied” or “extremely satisfied” categories.

Nearly all of the skills listed on each of the three checklists were marked as “Yes” (i.e., the student did perform this item). Of the 15 checklist items marked as a “No” (i.e., student did not perform this item), 73% were from the physical exam checklist. Two physical exam checklist items in particular (“Washed hands, or used sterilizer, before or after the physical exam” and “Conducted the physical exam from the patient's right”) accounted for 60% (n = 9) of the number of checklist items marked as “No”. Global ratings of students included scores of 3 or “meets expectations” (7%), 4 (37%) and 5 or “exceeds expectations” (56%) on the 1–5 Likert scale.

Correlations between various DOCS measures and clerkship evaluations of student performance are presented in Table 3. DOCS measures included the global rating from each of the 3 forms (interviewing, physical examination, and oral presentation) in addition to a “Summary Global Rating” that combined all global ratings for each student. A “Checklist Skills Score” was derived based on whether or not a student received a “No” on any of the three checklists (interviewing, physical examination, oral case presentation) from the DOCS forms. Students who had one or more items from any of the checklists marked as “No” were categorized as “0”; students who did not have any checklist item marked as “No” were categorized as “1”. Clerkship evaluation components included faculty and resident ratings of student clinical skills, a “Summary Clinical Rating” that combines faculty and resident ratings, written examination score, and final clerkship grade.

**Table 3.** Correlation Coefficients^§^ of DOCS Measures with Clerkship Measures

	Clerkship Measures									

	*Mean Faculty Rating*		*Mean Resident Rating*		*Written Exam Score*		*Summary Clinical Rating*		*Final Grade*	
					
DOCS measures	Correlation Coefficient	P-value	Correlation Coefficient	P-value	Correlation Coefficient	P-value	Correlation Coefficient	P-value	Correlation Coefficient	P-value
Global Rating: Interviewing/ Communication (N = 50)	0.11	0.47	0.07	0.62	0.03	0.83	0.06	0.65	0.21	0.14
Global Rating: Physical Examination (N = 55)	0.36	0.01	0.29	0.03	−0.03	0.80	0.37	0.0005	0.37	0.006
Global Rating: Oral Presentation (N = 56)	0.31	0.02	0.03	0.84	0.03	0.80	0.20	0.13	0.29	0.03
Summary Global Rating (N = 45)	0.33	0.02	0.13	0.38	0.08	0.60	0.26	0.09	0.34	0.02
Checklist Skills Score (0,1)^*^(N = 54)	0.23	0.09	0.40	0.002	−0.03	0.82	0.30	0.03	0.23	0.09

^§^assessed by the Spearman rank correlation coefficient.

^*^0 = at least one skill marked ‘NO’; 1 = all skills marked ‘YES’.

Overall, the relationships between the DOCS global ratings and clerkship measures of student clinical skills proved to be more statistically significant than the relationship between the DOCS Checklist Skills Score and clerkship measures of student clinical skills. There were no statistically significant associations between any DOCS measure and written examination scores.

Correlations between the DOCS physical exam global rating and each of the 3 clinical ratings (faculty, resident, and summary clinical ratings) and the final clerkship grade were all statistically significant. Both the DOCS oral presentation global rating and the Summary Global Rating were statistically significantly associated with faculty ratings and final clerkship grade. The relationship of DOCS global ratings to faculty ratings of student clinical skills was stronger than the relationship to resident ratings of student clinical skills.

For the DOCS Checklist Skills Score, only the statistical relationships with resident clinical ratings and the Summary Clinical Rating were significant. The relationship between Checklist Skills Score and final clerkship grade was marginal.

We also found a statistically significant relationship between the number of DOCS forms returned and the clerkship grade. As can seen in Table 4, students who did not return any forms were more likely to receive a “Pass,” while those who returned all 3 forms were more likely to receive “Honors.”

**Table 4.** Association between Number of Forms Returned and Final Clerkship Grade

	Final Clerkship Grade (N and %)			
		
Number of forms returned	Pass (P)	High Pass (HP)	Honors (H)	Total
0	9 (53%)	6 (35%)	2 (12%)	17 (100%)
1–2	3 (18%)	10 (59%)	4 (24%)	17 (100%)
3	6 (13%)	21 (47%)	18 (40%)	45 (100%)
Total	18 (23%)	37 (47%)	24 (30%)	79 (100%)

## Discussion

An ideal direct observation session for student clinical skills is feasible for both students and observers, provides a reliable and valid measure, correlates with other evaluation parameters or provides new information about student performance, and results in improved skills. We developed the DOCS session with these considerations and customized our program to meet the needs and structure of our curriculum.

Both the financial costs and the administrative burden of the DOCS sessions are relatively modest. The original process design incorporated DOCS into clerkship activities and clinical workflow. The DOCS forms themselves are fairly self-explanatory, and forms submission has been streamlined through the use of a self-addressed envelope attached to the forms and opportunities to submit the forms in locations where required clerkship sessions are scheduled. Limiting the DOCS exercise to one clinical site allowed a smaller group of observers to become more experienced with the session.

The majority of the DOCS observations occurred on-call or as part of clinical rounds and were completed within 30 minutes, suggesting that the sessions were time-efficient and integrated into clinical workflow The relatively high form return rate in the absence of any formal reminders to the students and the high satisfaction ratings from both students and evaluators also validate the DOCS program's feasibility.

The correlations between DOCS section scores and final clerkship grade are both instructive and encouraging. The strongest predictor of a high final grade was a high score in the DOCS physical examination section. Critics sometimes fault the assessment of student performance by residents and faculty as a “popularity contest” that rewards verbal facility more than actual clinical skills. If this indictment were correct, the predicted outcome would be a strong correlation between the DOCS case presentation score and the final clerkship grade. The positive but weaker correlation between these two variables tends to refute the criticism. The finding that the strongest correlation was between the DOCS score in physical examination and the final clerkship grade suggests that we are grading students on actual clinical skills.

The correlation between the DOCS case presentation score and the final clerkship grade reminds us that clinical teachers in Medicine, whether residents, ward attendings or teaching attendings, interact frequently with students in the setting of case presentation in rounds or seminars. Especially for the faculty, this form of interaction probably predominates over other potential interactions such as direct observations of patient encounters. A predictable result is that student's skills in case presentation would predominate in the teacher's assessment of his/her overall performance and that performance in case presentation would predominate in overall clinical performance assessment. Since performance assessment by residents and faculty constitutes a major portion of the grade in clerkships, stronger performance in case presentation would predictably be associated with higher final grades.

The correlation between case presentation scores and final grade would probably be strongest in clerkships that emphasize case presentation as part of the student's work. In addition, as the emphasis on case presentation probably varies among different medical colleges, so too might the correlation between case presentation scores and final grade.

The lack of correlation between DOCS scores and written examination scores supports the view that, as we had hoped, the two assessment measures evaluate distinct skill sets. Strong correlation would render the DOCS exercise redundant and unnecessary.

Although residents performed the majority of the DOCS observations, the DOCS scores correlated more highly with faculty evaluations of student performance than with resident evaluations of student performance. This lends construct validity to the DOCS exercises, which correlated more with the assessments of more experienced evaluators. Although DOCS sessions were only conducted at one clinical site, DOCS global ratings correlated with clinical ratings from multiple sites and observers, supporting concurrent validity with other clinical observations.

Not all students completed the DOCS exercises, and those who didn't received lower grades in the clerkship. This correlation may support the growing recognition that student “citizenship” in medical school is important. Indeed, an expanding body of literature has correlated signs of irresponsible behavior and inability to modify such behavior as a strong predictor for future professional misconduct and disciplinary action by state medical boards^10^.

A limitation of our study is that it was conducted during one clerkship at a single institution. Our current data about feedback and student and observer satisfaction only included quantitative information. Another potential limitation in interpreting the DOCS data is that attendings and residents have likely observed a student's clinical interactions during the clerkship prior to the DOCS assessment and may have preconceived ideas about the student's skills. In interpreting the correlation of DOCS measures and faculty/resident ratings of the students, it is also unclear whether prior interaction between a student and observer through the DOCS session impacted the clerkship rating.

Future areas of investigation of the utility of DOCS sessions include evaluation of generalizability to other clerkships and other institutions, collection of qualitative data related to student and observer satisfaction ratings of the session, additional assessment of concurrent validity by correlating DOCS results with other measures of student performance in clinical skills, and evaluation of reliability. The content and quality of the verbal feedback provided by observers, comparison of the feedback provided by residents as compared with attendings, and objective demonstration of students’ ability to use feedback from DOCS sessions in shaping their future performance in the domains of interviewing, physical examination, and oral presentation skills would also be important areas of future research.
